# Validating the pivotal role of the immune system in low‐dose radiation‐induced tumor inhibition in Lewis lung cancer‐bearing mice

**DOI:** 10.1002/cam4.1344

**Published:** 2018-02-25

**Authors:** Lei Zhou, Xiaoying Zhang, Hui Li, Chao Niu, Dehai Yu, Guozi Yang, Xinyue Liang, Xue Wen, Min Li, Jiuwei Cui

**Affiliations:** ^1^ Cancer Center The First Hospital of Jilin University Changchun 130021 China; ^2^ Department of Radiation‐Oncology The First Hospital of Jilin University Changchun 130021 China

**Keywords:** High‐dose radiation, immune enhancement, low‐dose radiation, lung cancer, mouse model

## Abstract

Although low‐dose radiation (LDR) possesses the two distinct functions of inducing hormesis and adaptive responses, which result in immune enhancement and tumor inhibition, its clinical applications have not yet been elucidated. The major obstacle that hinders the application of LDR in the clinical setting is that the mechanisms underlying induction of tumor inhibition are unclear, and the risks associated with LDR are still unknown. Thus, to overcome this obstacle and elucidate the mechanisms mediating the antitumor effects of LDR, in this study, we established an in vivo lung cancer model to investigate the participation of the immune system in LDR‐induced tumor inhibition and validated the pivotal role of the immune system by impairing immunity with high‐dose radiation (HDR) of 1 Gy. Additionally, the LDR‐induced adaptive response of the immune system was also observed by sequential HDR treatment in this mouse model. We found that LDR‐activated T cells and natural killer cells and increased the cytotoxicity of splenocytes and the infiltration of T cells in the tumor tissues. In contrast, when immune function was impaired by HDR pretreatment, LDR could not induce tumor inhibition. However, when LDR was administered before HDR, the immunity could be protected from impairment, and tumor growth could be inhibited to some extent, indicating the induction of the immune adaptive response by LDR. Therefore, we demonstrated that immune enhancement played a key role in LDR‐induced tumor inhibition. These findings emphasized the importance of the immune response in tumor radiotherapy and may help promote the application of LDR as a novel approach in clinical practice.

## Introduction

Nonsmall cell lung cancer (NSCLC) is the leading cause of cancer‐related death worldwide. The immunosuppressive state is a predominant feature that drives the progression and metastasis of cancer in patients with NSCLC receiving chemotherapy or radiotherapy 1. Moreover, the immune activity in patients with NSCLC at the clinically detectable stage is mostly suppressed 2, 3. Therefore, improving immune function is important for tumor regression in patients with NSCLC.

In contrast to high‐dose radiation (HDR), low‐dose radiation (LDR) has been proven to induce immune enhancement as a consequence of its hormesis effect 4, 5, 6. Radiation hormesis includes the effects of stimulating proliferation in normal cells, increasing lifespan, and enhancing fertility. LDR also induces adaptive responses that protect normal tissues around the tumors from impairment caused by subsequent HDR treatment resulting in increased antioxidant activity and DNA repair capacity, cell cycle redistribution, and susceptibility to apoptosis 4, 7. However, these radiation hormesis and adaptive responses do not exist in malignant cells 5, 8. Therefore, LDR could be exploited as a synergistic measure in tumor radiotherapy.

The hormesis response in the immune system induced by LDR has been extensively studied. LDR can stimulate both innate and adaptive immune responses 9. Our previous study showed that LDR can enhance the expansion and cytotoxicity of natural killer (NK) cells in vitro and increase the levels of interferon (IFN)‐*γ* and tumor necrosis factor (TNF)‐*α* in NK culture supernatants 10. Moreover, LDR can activate dendritic cells and augment T‐cell activation, thereby increasing antibody secretion and enhancing the antibody‐dependent cell‐mediated cytotoxicity response, which may shift naïve helper T cells to Th1 cells 9, 11. In this process, responses of T cells to antigens and mitogens are augmented 12 with increased cytokine (IFN‐*γ*, interleukin [IL]‐2) production, and the number of IL‐2 receptors on the surface of the T cells is also increased 13. The immunosuppressive regulatory T‐cell (Treg) population is reduced after LDR exposure in tumor‐bearing mice, which may reduce tumor burden and prolong survival 14, 15.

The adaptive response of LDR has also been found in the immune system. For example, total‐body LDR exposure induces the adaptive responses of thymocyte apoptosis and cell cycle progression in mice 16. In vivo LDR treatment protects human B lymphoblasts from subsequent HDR‐induced cell death 17, and high‐dose *γ*‐radiation‐induced DNA damage could be reduced in human peripheral blood mononuclear cells pretreated with low‐dose *γ*‐radiation 18.

These observations suggest that LDR may induce antitumor immunity. However, direct evidence demonstrating that immune augmentation is triggered by LDR and contributes to tumor regression is lacking. Thus, an in vivo tumor‐bearing model in normal mice, but not immunodeficient mice, is essential for investigating the role of immunity in LDR‐induced tumor regression. Accordingly, in this study, we established a model in normal C57BL/6 mice that exhibited Lewis lung cancer tumors; the mice received either total‐body LDR or HDR exposure only or sequential exposure to HDR before or after LDR. In this study, HDR was used to destroy the immune function of tumor‐bearing mice; HDR pretreatment before LDR was performed to inversely verify the immune‐enhancing effect of LDR, and LDR pretreatment before HDR was carried out to study the adaptive response of the immune system induced by LDR. The results indicated that LDR‐induced tumor inhibition was dependent on immune enhancement and that the adaptive response of the immune system could be induced by LDR, thereby protecting mice from HDR‐induced immune impairment.

## Materials and Methods

### Cells and cell lines

C57BL/6 mouse‐derived Lewis lung cancer cells were purchased from the American Type Culture Collection and cultured in Iscove's modified Dulbecco's medium (Gibco, Grand Island, NY) supplemented with 10% fetal bovine serum (FBS; Gibco) and penicillin–streptomycin antibiotics (Invitrogen, Carlsbad, CA). Mouse splenocytes were collected aseptically from spleens of mice by mincing the spleen tissues in a sterile Petri plate, and the erythrocytes were lysed in lysis buffer (10 mmol/L KHCO_3_, 150 mmol/L NH_4_Cl, 10 mmol/L ethylenediaminetetraacetic acid, pH 7.4). Separated splenocytes were cultured in Roswell Park Memorial Institute (RPMI) 1640 medium (Gibco) supplemented with 10% FBS and penicillin–streptomycin antibiotics.

### Mice

Female C57BL/6 mice were purchased from the Experimental Animal Center at Norman Bethune Medical College at Jilin University. Mice were housed in a specific pathogen‐free facility. The experimental manipulation of mice was performed according to the National Institute of Health Guide for the Care and Use of Laboratory Animals, with the approval of the Scientific Investigation Board of Science and Technology of Jilin Province.

### Tumor model

The mouse lung cancer model was established using previously reported methods 19. Briefly, 6‐ to 8‐week‐old female C57BL/6 mice were inoculated subcutaneously with 1 × 10^6^ Lewis lung cancer cells in 0.2 mL serum‐free medium at the right back near hind leg on day 0. Then, the mice were allocated randomly into five groups; in each group, 10 mice were used for tumor volume and survival analyses, and another 12 mice were used for splenocyte isolation at four time points. Tumor volume (length × width^2 ^× 0.5, in cubic millimeters) was measured every other day once tumors could be observed visually (after day 10). Animal survival was monitored after tumor inoculation.

### Radiation treatment

The mice were subjected to total‐body irradiation using an X‐ray generator (X‐RAD320UMSU). LDR at 75 mGy with a dose rate of 12.5 mGy/min and HDR at 1 Gy with a dose rate of 1 Gy/min were applied in this study. Four cycles of treatment were carried out in each group of tumor‐bearing mice. Briefly, mice in the LDR group received LDR treatment on days 10, 14, 18, and 22 after tumor inoculation; mice in the HDR group received HDR treatment on days 11, 15, 19, and 23; mice in the LDR‐HDR (LDR pretreatment 1 day before HDR) group received LDR on days 10, 14, 18, and 22 and received HDR on days 11, 15, 19, and 23; and mice in the HDR‐LDR (HDR pretreatment 1 day before LDR) group received HDR on days 9, 13, 17, and 21 and LDR on days 10, 14, 18, and 22. Mice in the Sham group were treated similarly except for the radiation. The animal experiment was replicated two times, and representative results are shown.

### Splenocyte proliferation assay

Three tumor‐bearing mice in each group were sacrificed on days 12, 16, 20, and 24 to isolate splenocytes. The proliferation of splenocytes was detected by WST‐1 assays. Briefly, splenocytes were resuspended to a concentration of 2 × 10^6^ cells/mL in RPMI 1640 with 10% FBS and seeded in 96‐well plates. Then, cells were treated with or without concanavalin A (Con A) at a final concentration of 5 *μ*g/mL. After incubation for 48 h at 37°C in an atmosphere containing 5% CO_2_, 20 *μ*L WST‐1 solution (Roche, Mannheim, Germany) was added, and incubation was resumed for an additional 3 h. Then, the plate was shaken for 20 sec and read at 440 nm using a microplate system (BioTek, Winooski, VT, US). Five replicate wells were read. The splenocyte proliferation index was calculated using the following formula: Proliferation index = (OD of the ConA group – OD of the control group/OD of the control group) × 100%.

### Cytokine measurement

The supernatants of splenocytes incubated with ConA were collected after 48 h of culture, and the levels of IL‐1*β*, IL‐2, IFN‐*γ*, TNF‐*α*, and IL‐10 were measured using a mouse cytometric bead array (CBA) kit (BD Biosciences, Bedford, MA, US). Briefly, 50 *μ*L of samples or standard samples (0–5000 pg/mL) were added to a mixture of 50 *μ*L each of capture antibody bead reagent and PE‐conjugated detection antibody. The mixture was then incubated for 2 h at room temperature in the dark and washed to remove unbound detection antibody. Data were acquired using a FACSCalibur flow cytometer (BD Biosciences) and analyzed using CBA software 1.1 (BD Biosciences).

### Splenocyte cytotoxicity assay

Target Lewis cells (1 × 10^6^) were labeled with 1 *μ*mol/L calcein AM and incubated for 30 min at 37°C. The labeled cells were then washed twice in phosphate‐buffered saline (PBS) and resuspended to 5 × 10^4^ cells/mL in RPMI 1640 with 5% FBS. Subsequently, 5 × 10^3^ labeled target cells in a 100‐*μ*L volume were plated in 96‐well plates, and mouse splenocytes were added as effector cells to an effector:target (E:T) ratio of 50:1. Cells were then incubated for 4 h at 37°C in an atmosphere containing 5% CO_2_. Next, 100 *μ*L of the supernatant was collected from each well and added to a new 96‐well plate, and the absorbance was measured using a microplate system (BioTek). The excitation wavelength was 485 nm, and the emission wavelength was 528 nm. Spontaneous release was determined by incubating target cells in medium alone, and maximum release was determined by suspending cells with 0.21% Triton X‐100. Splenocyte cytotoxicity was calculated using the following formula: killing efficiency (%) = (experimental release – spontaneous release)/(maximum release – spontaneous release) × 100.

### Flow cytometry

Freshly isolated splenocytes (2 × 10^5^) were stained with peridinin–chlorophyll–protein complex (PerCP)‐labeled anti‐CD3*ε* monoclonal antibodies (mAbs), fluorescein isothiocyanate (FITC)‐labeled anti‐CD4 mAbs, phycoerythrin‐labeled anti‐CD8a mAbs, allophycocyanin‐labeled anti‐NK1.1 mAbs, or FITC‐labeled anti‐CD69b mAbs (BD Biosciences) and incubated for 15 min in the dark at 25°C. The cells were then washed with PBS and analyzed using a FACSCalibur flow cytometer (BD Biosciences). The data were analyzed using FlowJo 7.6 (FlowJo LLC) software.

### Histopathological and immunohistochemical analyses

On day 24 after tumor inoculation, three mice in each group were sacrificed, and the tumors were removed. The tumor tissues were then fixed in 4% (w/v) formaldehyde, embedded in paraffin, sectioned, and stained with hematoxylin and eosin (H&E). For immunohistochemical analysis, the sections were deparaffinized, followed by antigen retrieval and blocking. The sections were then stained with anti‐mouse CD3 mAbs (1:500 dilution; Abcam, Cambridge, MA, US). The histopathological and immunohistochemical sections were observed using a microscope (Olympus, Model BX51TF, America, Inc.).

### Statistical analysis

Tumor growth curves were plotted based on tumor size until mouse death. Differences in tumor size among the various groups were determined by analysis of variance repeated measures test. Statistical significance for the survival of mice was assessed using Kaplan–Meier curves. Unpaired two‐tailed *t*‐tests were applied to analyze cell proliferation, cytotoxicity, and cytokine measurements. Differences with *P* values of less than 0.05 were considered statistically significant. Statistical analyses were performed using SPSS (IBM) and GraphPad Prism (GraphPad Software, Inc.) software.

## Results

### Tumor growth and survival in radiation‐exposed mice

**Figure 1:**
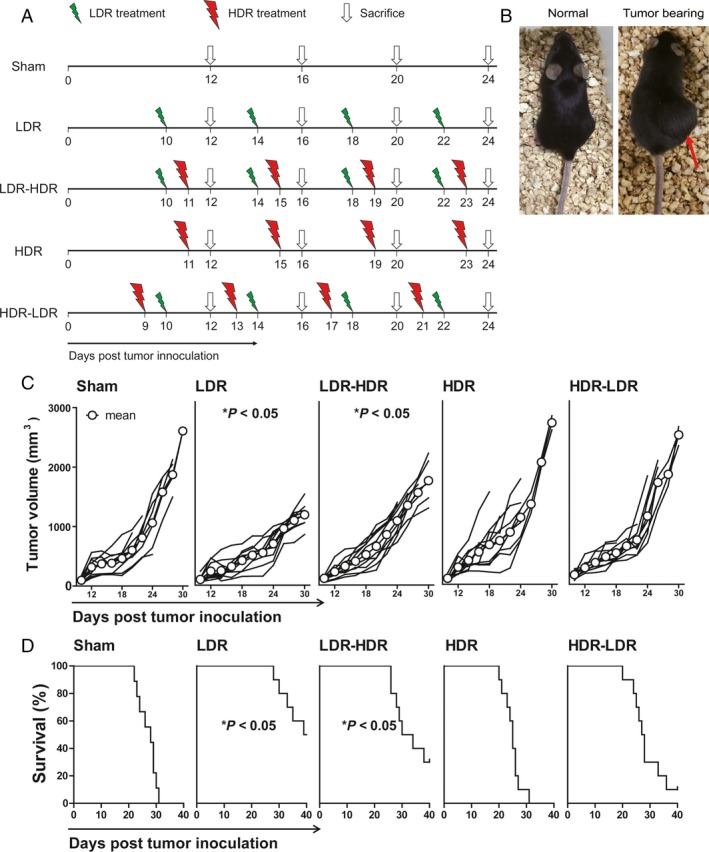
Tumor volume and survival of tumor‐bearing mice. C57BL/6 mice were inoculated with Lewis lung cancer cells on day 0 and received four cycles of treatment with 75 mGy LDR or 1 Gy HDR from day 9 to day 23. The tumor volumes of the mice were measured every 2 days, and the survival of the mice was calculated. (A) The protocol for radiation treatment. (B) Photograph of a normal mouse and a typical tumor‐bearing mouse. The red arrow represented the tumor site. (C) Tumor growth curves. Each line represents tumor growth kinetics in each mouse. (D) Survival curves of mice in each group. *P <* *0.05 versus Sham.

To observe the effects of LDR and HDR on tumor growth, female C57BL/6 mice were inoculated with Lewis lung cancer cells at 1 × 10^6^/mouse on day 0 and exposed to LDR or HDR as described above. The tumor volumes of the mice were measured every other day from day 10. As shown in Figure 1, the tumor volume data revealed that LDR treatment significantly inhibited tumor growth (*P *<* *0.05 vs. Sham; Fig. 1A) and prolonged survival of the mice (Fig. 1B). Notably, pretreatment with LDR before HDR significantly inhibited tumor growth and prolonged the survival of mice as well. Although the kinetic curves of tumor growth in the HDR and HDR‐LDR groups were similar to those in the Sham group, the survival of mice in these two groups did not improve, indicating that these two treatment regimens could not inhibit tumor growth.

Analysis of the survival data (Fig. 1B) showed that LDR treatment prolonged the survival of tumor‐bearing mice; five of 10 mice lived to day 40. There were three of 10 mice in the LDR‐HDR group and one of 10 mice in the HDR‐LDR group that lived to day 40, whereas all mice in the Sham and HDR groups died by day 31. The median survival times were 28 (Sham), 39.5 (LDR), 32 (LDR‐HDR), 25 (HDR), and 27.5 (HDR‐LDR) days. These results indicated that LDR and LDR pretreatment before HDR inhibited tumor growth and prolonged survival in Lewis lung cancer‐bearing mice, but HDR and HDR pretreatment before LDR did not induce tumor inhibition.

### LDR increased the mitogen response of splenocytes in tumor‐bearing mice

**Figure 2:**
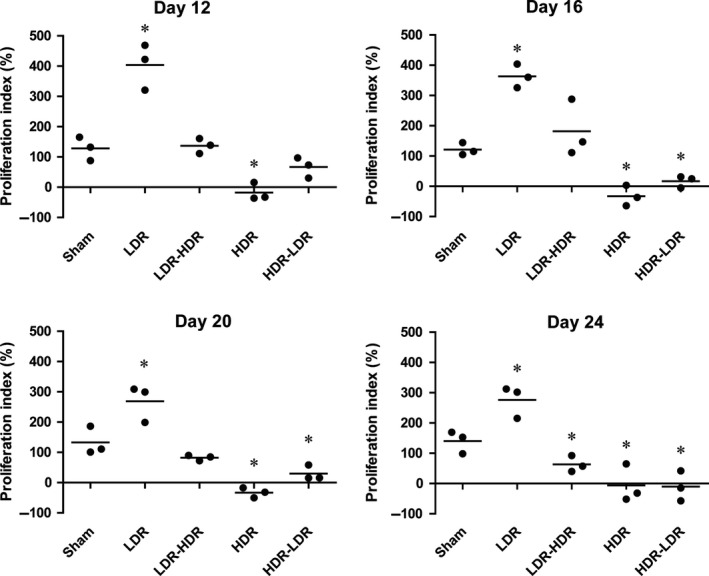
Splenocyte response to mitogen stimulation. Three tumor‐bearing mice in each group were sacrificed on days 12, 16, 20, and 24, and their splenocytes were isolated and cultured with or without 5 *μ*g/mL ConA for 48 h. Splenocyte proliferation was detected by WST‐1 assays, and the proliferation index was calculated. *P <* *0.05 versus Sham.

On days 12, 16, 20, and 24, three tumor‐bearing mice in each group were selected randomly and sacrificed, and their spleens were isolated to examine the mitogen responses of splenocytes induced by ConA. The splenocytes were incubated with 5 *μ*g/mL ConA for 48 h, and WST‐1 assays were carried out to determine the splenocyte mitogen response, after which the proliferation index was calculated. The results (Fig. 2) showed that the splenocytes of the mice that received LDR exhibited the highest mitogen response on day 12, 16, 20, and 24; while compared with Sham and LDR, HDR impaired the splenocyte mitogen response or even inhibited splenocyte proliferation after ConA stimulation. Moreover, the results also showed that LDR pretreatment before HDR could protect splenocytes from HDR‐induced severe impairment; the splenocyte proliferation index of mice in the LDR‐HDR group was higher than that in the HDR group, and the mean value on day 16 was even higher than that in the Sham group. However, LDR treatment followed by HDR did not protect splenocytes from impairment from HDR, as the splenocyte proliferation index in the HDR‐LDR group was similar to or only slightly higher than that in the HDR group.

### LDR influenced cytokine production in mouse splenocytes

**Figure 3:**
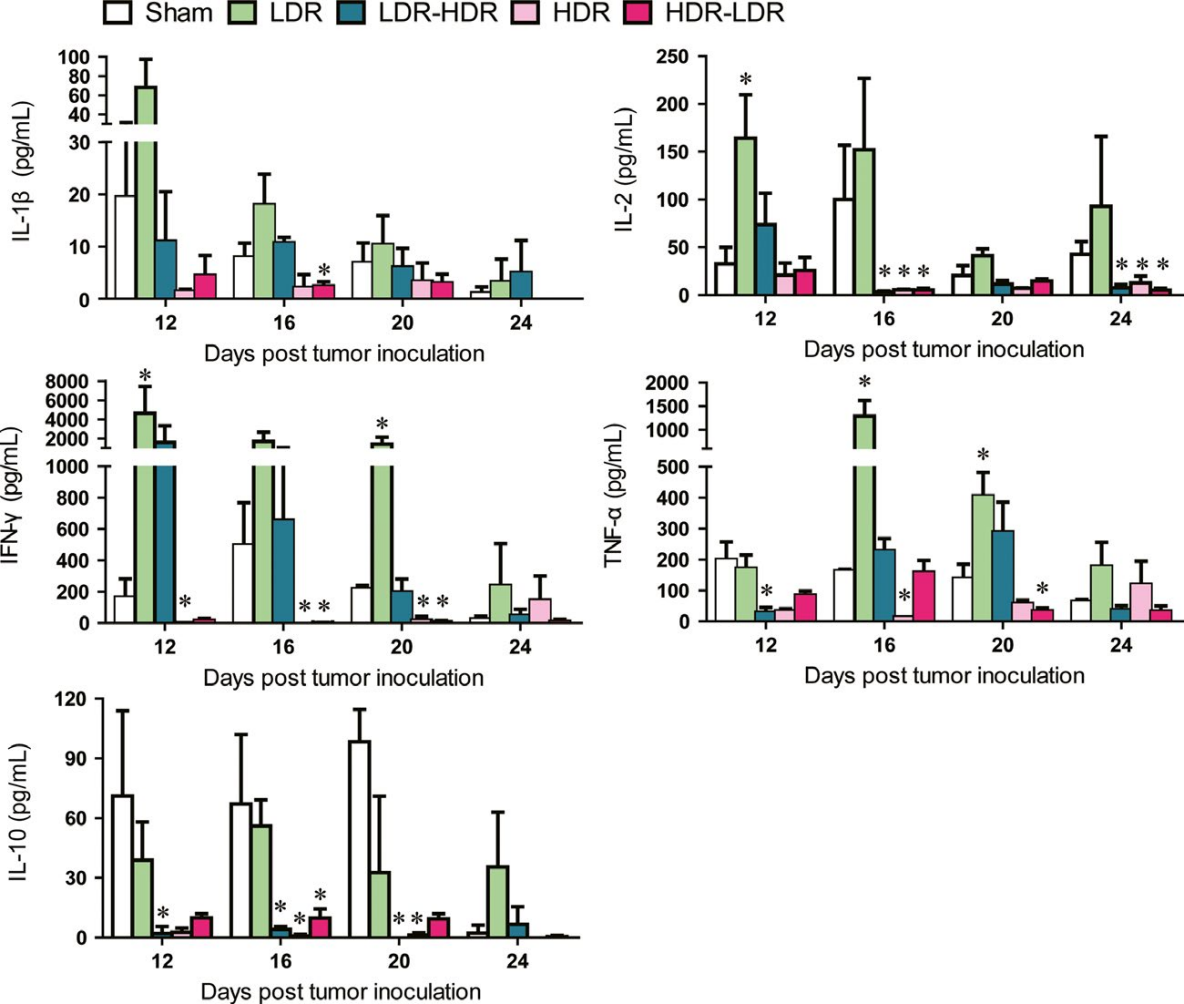
Cytokine production by mouse splenocytes. The supernatants of the cultured splenocytes were collected, and the levels of cytokines, including IL‐1*β*, IL‐2, IFN‐*γ*, TNF‐*α*, and IL‐10, were detected using a mouse CBA kit. *P <* *0.05 versus Sham.

After incubation with ConA for 48 h, supernatants of the splenocytes were collected, and IL‐1*β*, IL‐2, IFN‐*γ*, TNF‐*α*, and IL‐10 were detected. As shown in Figure 3, compared with the Sham group, LDR significantly or clearly induced upregulation of the Th1 cytokines IL‐1*β*, IL‐2, IFN‐*γ*, and TNF‐*α* and downregulated IL‐10 production on days 12, 16, and 20, whereas HDR inhibited the production of these cytokines. LDR pretreatment protected the cytokine‐producing ability of splenocytes on days 12, 16, and 20 to some degree; however, this effect did not last up to day 24. In contrast, compared with HDR treatment, HDR‐LDR treatment did not exhibit protective effects on cytokine production.

### LDR‐activated T cells and NK cells

**Figure 4:**
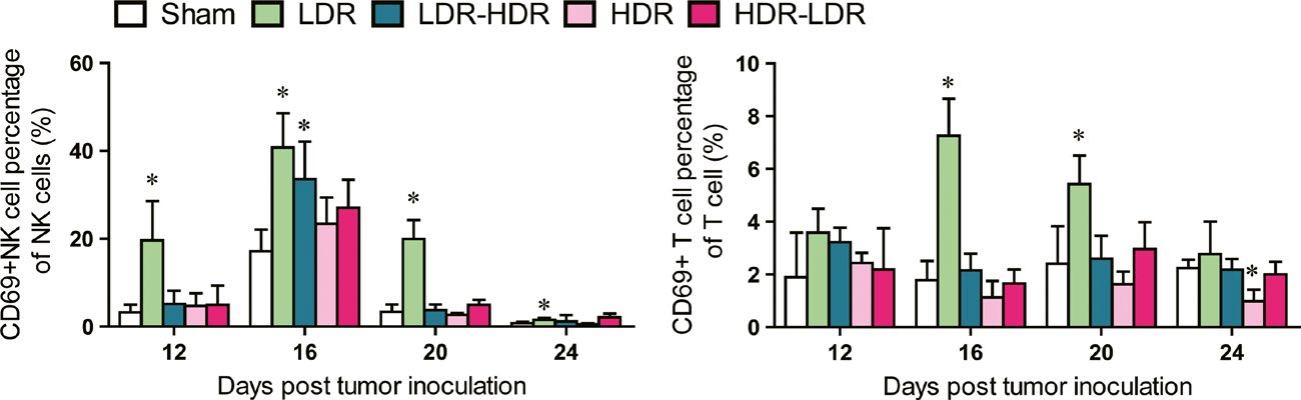
Percentages of CD69^+^ NK cells and T cells in mouse spleens. The CD69^+^ NK cells in splenocytes were identified by staining splenocytes with anti‐CD3, anti‐NK1.1, and anti‐CD69 mAbs, and the percentage of CD3^−^/NK1.1^+^/CD69^+^ cells in CD3^−^/NK1.1^+^ cells was calculated. The CD69^+^ T cells were identified by staining splenocytes with anti‐CD3 and anti‐CD69 mAbs, and the percentage of CD3^+^/CD69^+^ cells in CD3^+^ cells was calculated. *P <* *0.05 versus Sham.

Because CD69 is an early activation molecule expressed on the surface of cytotoxic leukocytes, we detected CD69 expression in splenic NK cells and CD8^+^ T cells. As shown in Figure 4, the percentage of CD69^+^ NK cells in mice in the LDR group was significantly higher than that in the Sham group on days 12, 16, 20, and 24, and the percentage of CD69^+^ CD8^+^ T cells in the LDR group was significantly higher than that in the Sham group on days 16 and 20. Similarly, on day 16, the percentage of CD69^+^ NK cells in the LDR‐HDR group was significantly higher than that in the Sham group. However, HDR treatment significantly decreased the percentage of CD69^+^ CD8^+^ T cells on day 24.

### LDR increased cytotoxicity of splenocytes

**Figure 5:**
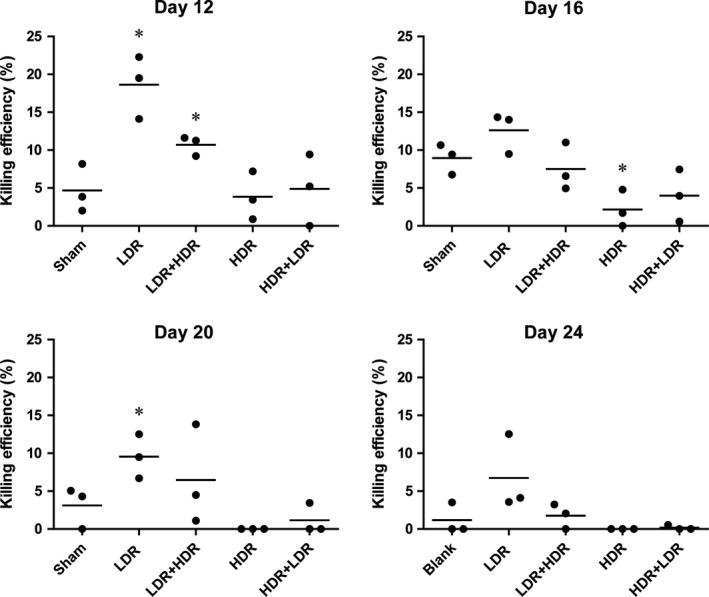
Cytotoxicity of mouse splenocytes. The splenocyte cytotoxicity of the tumor‐bearing mice was determined by incubation with calcein AM‐labeled Lewis lung cancer cells, with an E:T of 50:1. The absorbance of the supernatants was detected, and the killing efficiency was calculated. *P <* *0.05 versus Sham.

The splenocytes of tumor‐bearing mice were isolated and incubated with calcein‐stained Lewis lung cancer cells at an E:T ratio of 50:1 for 4 h, and the supernatants were then collected to detect calcein release. As shown in Figure 5, compared with Sham radiation, LDR effectively increased the cytotoxicity of splenocytes on days 12 and 20, and LDR‐HDR treatment also increased cytotoxicity on day 12. However, HDR treatment significantly inhibited cytotoxicity on day 16.

### Pathological changes and CD3^+^ T‐cell infiltration into tumors

**Figure 6:**
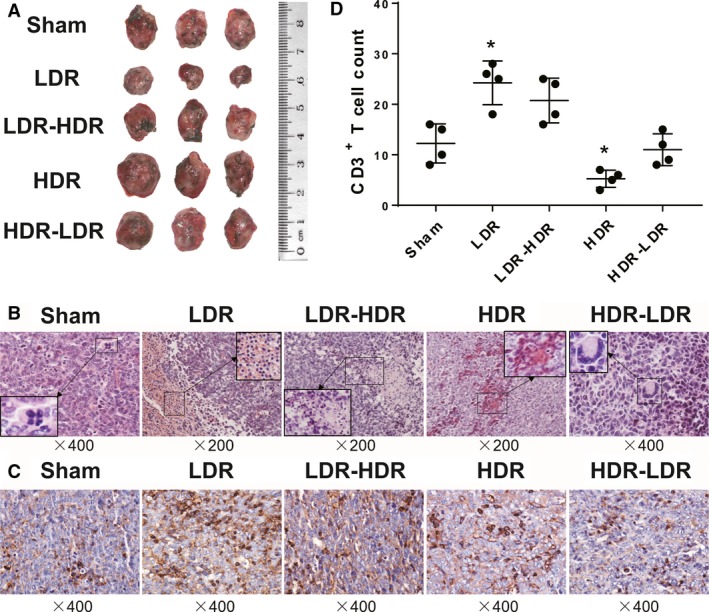
Pathological and immunohistochemical analysis. The tumors of the Lewis lung cancer‐bearing mice were isolated on day 24 after tumor inoculation. (A) Photographs of the tumors. The pathological sections were stained with H&E (B), and T‐cell infiltration identification in tumor tissues was conducted by CD3 staining (C). CD3^+^ cells were quantified by counting randomly selected fields (D). Representative images are shown. *P <* *0.05 versus Sham.

Pathological examination was conducted on day 24 after tumor inoculation. Three mice in each group were killed, and the tumors were isolated and sectioned. The sections were then stained by H&E and anti‐CD3 antibodies for immunohistochemical analysis. As shown in Figure 6A, massive tumor cell death was observed in the LDR and LDR‐HDR groups, and tumor cell degeneration was induced in the HDR group. Cell division and multinucleated giant cells were observed frequently in the Sham and HDR‐LDR groups, indicating the occurrence of vigorous DNA replication. In immunohistochemical analysis of CD3 staining, many infiltrated T cells were observed in tumor tissues of mice in the LDR and LDR‐HDR groups, and fewer CD3^+^ cells infiltrated in the Sham, HDR, and HDR‐LDR groups, suggesting that LDR could enhance the antitumor immune response in tumor‐bearing mice.

## Discussion

In this study, a xenograft model was established in normal mice. The advantage of this in vivo model was that it allowed us to explore the participation of the immune system in LDR‐induced tumor inhibition. The LDR‐induced adaptive response of the immune system was also observed through immune impairment induced by subsequent HDR treatment.

LDR is known to be associated with the ability to enhance innate and adaptive immunity in tumor‐bearing animals. The data in this study provide direct evidence demonstrating that total‐body LDR exposure can enhance antitumor immunity and inhibit tumor progression in mice, accompanied by induction of adaptive responses in the immune system. Our results demonstrated that the antitumor immunity induced by LDR was mainly related to NK cell and T‐cell activation, as manifested by increased expression of CD69. This was consistent with the results of our previous work showing that LDR augmented the cytotoxicity of NK cells in vitro 10 through increased production of IFN‐*γ*, TNF‐*α*, perforin, and granzymes 20, 21. An in vivo study also reported that a single low dose of X‐ray irradiation inhibited tumor metastases and triggered NK cell activity in BALB/C mice with L1 sarcoma. Therefore, the role of NK cells in LDR‐induced tumor inhibition could be determined. T cells are also involved in the immune enhancement induced by LDR, as manifested by augmented proliferation and response to antigenic and mitogenic stimulation and cytokine production 12, 22, particularly production of IFN‐*γ* and IL‐2 23. Coincidently, in the current study, we found a similar T‐cell reaction induced by LDR, including upregulation of CD69 molecules in T cells, increased cytotoxicity of mouse splenocytes, and increased infiltration of T cells in the tumor tissues. Remarkably, an increase in Th1 cytokine production (IFN‐*γ*, IL‐2, TNF‐*α*, and IL‐1*β*) and a concomitant decrease in Th2 cytokine production (IL‐10) were detected in the splenocyte supernatants of LDR‐treated mice. The switch from Th2 to Th1 cytokine production may be related to the antitumor effects of LDR. We also found that compared with the data for days 12 and 14, LDR‐induced antitumor immunity decreased on day 20 and thereafter, particularly for killing assays and CD69^+^ NK cell and T‐cell percentages, although total survival and tumor growth were improved. Tumors are known to induce immune suppression through multiple mechanisms, facilitating their escape from immune surveillance. Therefore, we speculate that the decreased ability of LDR to induce antitumor immunity may have been related to tumor‐induced immune suppression. During the early stage, when the tumors were small in size, they caused only minor suppression of mouse immunity. Then, as the tumors grew larger after day 20, they caused greater immune impairment, decreasing the ability of LDR to induce antitumor immunity. Such speculation could be supported by a study in the same mouse model showing that generalized T‐cell exhaustion was induced by the tumor 3 weeks after tumor inoculation 24.

To further validate the involvement of immune enhancement in LDR‐induced tumor inhibition, HDR pretreatment was carried out before LDR treatment to impair the immune function as an inverse validation. The dose of HDR was 1 Gy, which was considered insufficient to directly inhibit tumor growth because the effective dose of radiation to treat Lewis lung cancer in mice is 13 Gy or more 23, 25, 26, 27. As expected, we found that HDR markedly impaired immune function, resulting in decreased splenocyte proliferation and cytokine production. When HDR pretreatment was administered before LDR, LDR could not induce splenocyte proliferation and cytokine production, and the tumor inhibition effect was not observed. The immunohistochemical analysis of CD3^+^ cell infiltration in tumor tissues also revealed the involvement of immunity in LDR‐induced tumor inhibition. These findings indicated that the antitumor effects of LDR were mediated by enhanced immunity.

The adaptive response of LDR to protect normal tissues from HDR lesions has been observed in many previous studies. For example, 5 cGy pre‐exposure increased the 30‐day survival rate from 30% to 70% in ICR mice exposed to 8 Gy X‐rays 28; low‐dose pre‐exposure delayed the onset of subsequent HDR exposure‐induced leukemia 29. The mechanisms of LDR in inducing an adaptive response were found to be related to stimulating the antioxidant response 30, 31, DNA damage repair 7, 32, 33, and modifying glucose metabolism 4, 34. However, in tumor‐bearing mice, we found that LDR could also induce an adaptive response in the immune system. Although total‐body exposure to 1 Gy HDR four times did not inhibit tumor growth, it did impair mouse immune function. However, we found that 75 mGy LDR pretreatment before 1 Gy HDR ameliorated the HDR‐induced immune impairment and finally tended to inhibit tumor growth and promote survival compared with the Sham and HDR treatment. Moreover, there was more infiltration of CD3^+^ cells in tumor tissues subjected to LDR pretreatment with HDR than in those subjected to HDR treatment alone. Although further studies are needed to elucidate the molecular mechanisms involved in these processes, these findings verified the adaptive response of LDR in the immune system.

In conclusion, the current work demonstrated that the immune system plays a pivotal role in LDR‐induced tumor inhibition, and LDR pretreatment is beneficial to tumor radiation therapy for enhancing antitumor immunity and ameliorating HDR‐induced immune impairment. This may substantially optimize the radiotherapy plan and provide a safer and more effective regimen. However, although our previous study demonstrated that LDR induced NK cell activation in vitro, most likely through the p38 mitogen‐activated protein kinase pathway, the molecular mechanisms of LDR‐induced antitumor immunity enhancement should be explored in greater detail in subsequent in vivo studies. Moreover, the risks of LDR, including the cumulative toxicity of repeated LDR exposure, must be further evaluated.

## Conflict of Interest

No potential conflict of interests was disclosed.
